# The Short-chain Fatty Acid Propionic Acid Activates the Rcs Stress Response System Partially through Inhibition of d-Alanine Racemase

**DOI:** 10.1128/msphere.00439-22

**Published:** 2023-01-16

**Authors:** Nathaniel S. Harshaw, Mitchell D. Meyer, Nicholas A. Stella, Kara M. Lehner, Regis P. Kowalski, Robert M. Q. Shanks

**Affiliations:** a Department of Ophthalmology, University of Pittsburgh School of Medicine, Pittsburgh, Pennsylvania, USA; b Department of Microbiology and Molecular Genetics, University of Pittsburgh School of Medicine, Pittsburgh, Pennsylvania, USA; The University of Iowa

**Keywords:** propionic acid, alanine racemase, short-chain fatty acid, Rcs system, stress response, Enterobacterales, antibiotic tolerance

## Abstract

The Enterobacterial Rcs stress response system reacts to envelope stresses through a complex two-component phosphorelay system to regulate a variety of environmental response genes, such as capsular polysaccharide and flagella biosynthesis genes. However, beyond Escherichia coli, the stresses that activate Rcs are not well-understood. In this study, we used a Rcs system-dependent luminescent transcriptional reporter to screen a library of over 240 antimicrobial compounds for those that activated the Rcs system in Serratia marcescens, a *Yersiniaceae* family bacterium. Using an isogenic *rcsB* mutant to establish specificity, both new and expected activators were identified, including the short-chain fatty acid propionic acid, which is found at millimolar levels in the human gut. Propionic acid did not reduce the bacterial intracellular pH, as was hypothesized for its antibacterial mechanism. Instead, data suggest that the Rcs-activation by propionic acid is due, in part, to an inactivation of alanine racemase. This enzyme is responsible for the biosynthesis of d-alanine, which is an amino-acid that is required for the generation of bacterial cell walls. Consistent with what was observed in S. marcescens, in E. coli, alanine racemase mutants demonstrated elevated expression of the Rcs-reporter in a d-alanine-dependent and RcsB-dependent manner. These results suggest that host gut short-chain fatty acids can influence bacterial behavior via the activation of the Rcs stress response system.

**IMPORTANCE** The Rcs bacterial stress response system responds to envelope stresses by globally altering gene expression to profoundly impact host-pathogen interactions, virulence, and antibiotic tolerance. In this study, a luminescent Rcs-reporter plasmid was used to screen a library of compounds for activators of Rcs. Among the strongest inducers was the short-chain fatty acid propionic acid, which is found at high concentrations in the human gut. This study suggests that gut short-chain fatty acids can affect both bacterial virulence and antibiotic tolerance via the induction of the Rcs system.

## INTRODUCTION

The regulation of capsule synthesis among Enterobacterales species is performed by the Rcs system, which is a highly conserved bacterial stress response system. The Rcs pathway relies on complex phosphorelay signal transduction through the interplay of multiple inner and outer membrane components, including RcsC, RcsD, RcsF, IgaA, RcsB (a response regulator), and RcsB-binding accessory proteins, including RcsA (1–3). RcsF interacts with the outer membrane proteins OmpA, OmpC, and OmpF to monitor outer membrane integrity (4). Together, these components are responsible for the transcriptional regulation of genes induced by cell wall and outer membrane stresses, the scope of which is not fully understood. Known Rcs system activators include mutations, enzymes, peptides, and drugs that affect the outer membrane either directly or via the inhibition of the enzymes that are necessary for outer membrane biogenesis (4–8). In addition, mutations, enzymes, or drugs that target peptidoglycan synthesis can activate the Rcs system (7, 9–12), as can osmotic and oxidative stress (13, 14).

Although Rcs is activated by many antibiotics, activation does not confer notably reduced antibiotic susceptibility. However, at near-minimal inhibitory antibiotic concentrations, Rcs activation promotes bacterial survival, indicating that activation could increase bacterial persistence host niches with near-inhibitory antibiotic levels that do not achieve sufficient levels to inactivate them (12). The Rcs system is also of interest as a regulator of virulence determinants, including capsules, secreted toxins, flagella, and adhesion molecules (15–19). Mutations that activate or inactivate the Rcs system confer altered virulence in diverse animal models (20–22). Therefore, it is likely that the regulation of the Rcs system is critical to bacterial survival during infections.

For this study, S. marcescens was used as a model organism. S. marcescens is an opportunistic pathogen of the family *Yersiniaceae* that causes contact lens-associated keratitis in healthy patients as well as nosocomial infections, such as ventilator-associated pneumonia, in the immunocompromised. S. marcescens is associated with microbial dysbiosis in Crohn’s disease (23), and it is found in numerous environmental niches, such as water, soil, plants, coral, and the gut flora of mammals and insects (24, 25). S. marcescens mutants with an inactivated or overactivated Rcs system demonstrate shifts in the global transcriptional landscape (19, 26). Other work has demonstrated a role for the Rcs system in the regulation of S. marcescens virulence and virulence factors, including the ShlA cytolysin (27). In S. marcescens, the Rcs system can be activated by mutations that affect enterobacterial common antigen synthesis and by the mutation of the S. marcescens IgaA ortholog, GumB (28, 29). The antibiotics that are used for the treatment of ocular infections activate the S. marcescens Rcs system, and this includes antibiotics to which the bacteria are susceptible (ceftazidime) and resistant (cefazolin, polymyxin B, and vancomycin). Additionally, the Rcs system regulates horizontal gene transfer and the phage defense systems of *Serratia* species (30).

In this study, a luminescence reporter for Rcs system activation was used to screen a library of over 240 compounds to elucidate the breadth of insults that the Rcs system detects. This was used in the wild-type (WT) S. marcescens and in a Rcs-deficient mutant to establish Rcs system specific effects. The ability of one of the most abundant gut short-chain fatty acids (SCFA), namely, propionic acid, to specifically activate the Rcs system was identified and evaluated. Propionic acid, which is produced by the microbiota through the digestion of dietary fiber, is found at high concentrations in the human gut (up to 15 to 26 mM) and blood (up to 75 μM) (31–33). Hence, it likely impacts the behavior of bacteria via the Rcs or similar stress response systems.

## RESULTS

### Screen for chemical activators of the Rcs system.

**FIG 1:**
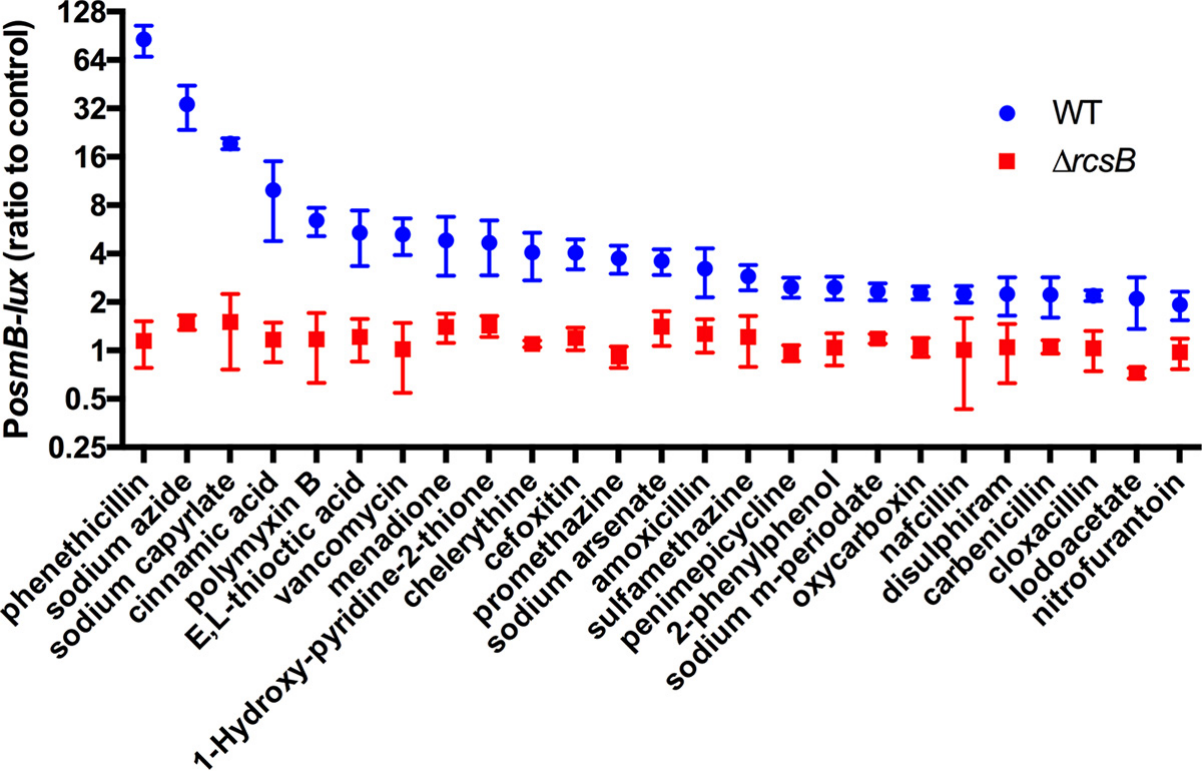
Compounds that activate an Rcs system-regulated promoter in S. marcescens. A luminescent reporter for the Rcs system (P*_osmB_*) was used to screen for compounds that activate the Rcs system from Biolog Phenotypic Microarray Plates PM11-20. Only compounds that elicited a twofold or greater difference between the WT and Δ*rcsB* are shown. *P* < 0.05 by Student’s *t* test. The mean and standard deviation values are shown. *n* = 3.

A plasmid-borne luminescence reporter construct was used to screen for molecules that activate the Rcs system (Table 1; Fig. S1, Supplemental Data Set S1). The previously described plasmid pMQ747 includes the promoter for the SMDB11_1637 open reading frame, which is induced by the Rcs system in S. marcescens as well as in other bacterial genera and is predicted to code for the conserved *osmB* gene (29, 34, 35). A clinical isolate of S. marcescens bearing a plasmid with this reporter, pMQ747, was grown in lysogeny broth (LB) medium and added to Biolog Phenotypic Microarray Plates PM11-20. These 96-well plates contained over 200 compounds, many of which have antimicrobial effects. These compounds were then categorized partly based on work by Dunkley et al. (36) (Data Set S1). Each compound was replicated in 3 to 4 different proprietary concentrations. The bacteria were added to wells at a specified culture density and grown for 4 h, at which point the optical density and luminescence were measured. The 4 h time point was chosen, based empirically on prior experiments that found it to be optimal (29). As a control, the culture density was measured at the initial time point to identify the compounds that altered the optical density independently of the bacterial growth. To test whether the effects were specific to the Rcs system, the pMQ747 *P_osmB_* reporter plasmid was transformed into a Δ*rcsB* mutant that lacked the Rcs response regulator, RcsB. The compounds that induced a twofold or higher expression in the WT, compared to the Δ*rcsB* strain, were considered to activate the reporter in an Rcs-dependent manner. A heat map of all of the tested compounds demonstrates clear differences between the WT and Δ*rcsB* mutant as well as between the compound groups (Fig. S1). 25 compounds were found to increase expression by twofold or higher in the WT than in the control and were expressed at significantly higher levels than those observed in the Δ*rcsB* mutant (Fig. 1). These compounds include known activators of the Rcs system, such as polymyxin B and vancomycin, that were previously shown to activate this reporter (29) as well as several novel Rcs-activating compounds. Notable among these were the medium-chain fatty acid sodium caprylate and the sodium salt of octanoic acid, which induced an almost 20-fold increase in luminescence (Fig. 1).

**TABLE 1:** Bacterial strains and plasmids used in this study

Strain/Plasmid	Description	Source
K904	Wild-type S. marcescens keratitis isolate	76
CMS5177	Δ*rcsB*, K904 with *rcsB* ORF replaced by *mClover*	72
CMS4133	K904 with *rcsC* insertion mutation	20
MG1655	E. coli K-12 F- λ- *ilvG- rfb-50 rph-1*	53, Coli Genetic Stock Center
MB2159	E. coli *alr::frt dadX::frt*	52, Coli Genetic Stock Center
MB2159a	MB2159 spontaneous d-alanine prototroph	This study
CMS5702	MB2159 with *ΔrcsB::kan*	This study
pMQ414	oriRSF1010-based plasmid with P*nptII*-gfp	73
pMQ747	pMQ713 with SMDB11_1637 (*osmB*) promoter-l*uxCDABE*	29
pMQ748	pMQ713 with SMDB11_2817 promoter-l*uxCDABE*	29
pMQ749	pMQ713 with *umoD* promoter –*luxCDABE*	29
pMQ802	pMQ414 with pHLuorin2 replacing *tdtomato*, codon optimized for S. marcescens	This study
pKD4	Source of kanamycin resistance marker	71
pMQ538	Lambda red recombineering plasmid	72

10.1128/msphere.00439-22.1 FIG S1 Heat map of compounds that activate the P*osmB-lux* Rcs-stress reporter. S. marcescens strains with the P*_osmB_*-*luxCDABE* reporter were grown in Biolog Phenotypic Microarray Plates PM11-20 and were evaluated for luminescence and optical density after 4 h of incubation at 30°C. The ratio of luminescence to growth (RLU) values were normalized to the RLU of the control wells with no compound to determine the fold induction. The highest fold value for each compound was used (each compound had 3 to 4 wells with different concentrations). The experiment was performed three times. Values were plotted using the Heatmapper software. Download FIG S1, TIF file, 0.5 MB. Copyright © 2023 Harshaw et al. 2023 Harshaw et al. https://creativecommons.org/licenses/by/4.0/ This content is distributed under the terms of the Creative Commons Attribution 4.0 International license.

10.1128/msphere.00439-22.7 DATA SET S1 An Excel file containing the RLU results for each compound. Download Data Set S1, XLSX file, 0.1 MB. Copyright © 2023 Harshaw et al. 2023 Harshaw et al. https://creativecommons.org/licenses/by/4.0/ This content is distributed under the terms of the Creative Commons Attribution 4.0 International license.

Biolog Gen III plates were also used with the WT only. The Gen III plate consists of a 96-well plate with different potentially stress-inducing compounds, such as sodium chloride and antibiotics, that are designed to differentiate bacterial species. Of these compounds, three fatty acids, sodium butyrate (2.9-fold), α-keto-butyric acid (2.4-fold), and propionic acid (2.0-fold) correlated with induced reporter activity in the WT, compared to the control (well with no compound) (Fig. S2).

10.1128/msphere.00439-22.2 FIG S2 Detection of chemicals that activate the P*osmB* using a Biolog Gen III plate. S. marcescens strains with a P*_osmB_*-*luxCDABE*-reporter for Rcs-dependent stress were introduced into a Biolog GenIII plate, incubated at 30°C, and measured for luminescence and optical density (OD_600_) at 4 and 6 h after inoculation. The relative induction values, compared to the control values, demonstrate the reproducible induction of the Rcs system by a number of chemicals. The mean and standard deviation values from 4 independent experiments are shown. The luminescence was normalized by the culture density and compared to the strain without a test chemical (control), which was set to a value of 1. Asterisks indicate statistically significant differences from the control as evaluated by an analysis of variance (ANOVA) with Dunnett’s multiple-comparison test. Download FIG S2, TIF file, 0.3 MB. Copyright © 2023 Harshaw et al. 2023 Harshaw et al. https://creativecommons.org/licenses/by/4.0/ This content is distributed under the terms of the Creative Commons Attribution 4.0 International license.

Unexpectedly, a group of compounds activated the reporter more highly in the Δ*rcsB* mutant (Table 2; Fig. S4A). We hypothesize that these compounds activate other stress response systems that are capable of regulating the *P_osmB_* promoter and are repressed by the Rcs system (Fig. S4B). These compounds include nucleic acid metabolism-targeting antibiotics, such as rifamycin SV, cinoxacin, and nalidixic acid. Two macrolide antibiotics that target translation, namely, tylosin and spiramycin, were also identified to increase reporter activation.

**TABLE 2:** Compounds that induce the *P_osmB_-luxCDABE* reporter more highly in the Δ*rcsB* mutant

Compound	Compound class	Function	Fold Δ*rcsB* compound/control^*a*^	Fold (Δ*rcsB*/WT)^*b*^	*P* value^*c*^
Rifamycin SV	Rifamycins	Nucleic acid synthesis inhibitor	35.4	8.4	0.001
Boric acid	Antiseptic, disinfectant	Antiseptic, disinfectants	23.8	13.5	0.033
Sodium metaborate	Metal ions	Possible oxidative metabolism inhibitor	20.9	8.6	0.003
Cinoxacin	Quinolone	Nucleic acid synthesis inhibitor	16.4	6.7	0.002
3,5-diamino-1,2,4-triazole (guanazole)	Cytostatic triazole	Nucleic acid synthesis inhibitor	12.0	6.1	0.037
Nalidixic acid	Quinolone	Nucleic acid synthesis inhibitor	8.6	2.5	0.045
DL-methionine hydroxamate	Amino acid analog	Metabolic compound/inhibitor	5.3	3.0	0.012
Tylosin	Macrolide	Protein synthesis inhibitor	4.4	3.6	0.026
Oxolinic acid	Quinolone	Nucleic acid synthesis inhibitor	4.3	2.0	0.007
Spiramycin	Macrolide	Protein synthesis inhibitor	4.8	3.5	0.038
Cefoxitin	Cephalosporin	Cell wall targeting	4.0	3.4	0.005
Promethazine	Medication/metabolic inhibitor	Repurposed medication	4.7	3.3	0.033
Sodium arsenate	General toxin, germicide	Other	2.1	2.9	0.032

a Average of the WT levels with compound over the WT levels in the control wells. *n* = 3.

b Average of the WT levels divided by the Δ*rcsB* mutant levels. *n* = 3.

c Student’s *t* test of the WT levels versus the Δ*rcsB* levels.

10.1128/msphere.00439-22.4 FIG S4 Compounds that activate P*_osmB_* more highly in the Δ*rcsB* mutant. (A) A luminescent reporter for the Rcs system was used to screen for compounds that activate the Rcs system from Biolog Phenotypic Microarray Plates PM11-20. Only the compounds that elicited a twofold or greater difference between Δ*rcsB* and the control and were statistically significantly different between the WT and Δ*rcsB* are shown. *P* < 0.05 by Student’s *t* test. The mean and standard deviation values are shown. *n* = 3. (B) Model for the Rcs inhibition of another stress response system (SRS) that in turn controls P*osmB*. Download FIG S4, TIF file, 0.3 MB. Copyright © 2023 Harshaw et al. 2023 Harshaw et al. https://creativecommons.org/licenses/by/4.0/ This content is distributed under the terms of the Creative Commons Attribution 4.0 International license.

### Propionic acid activates RcsB-regulated promoters.

**FIG 2:**
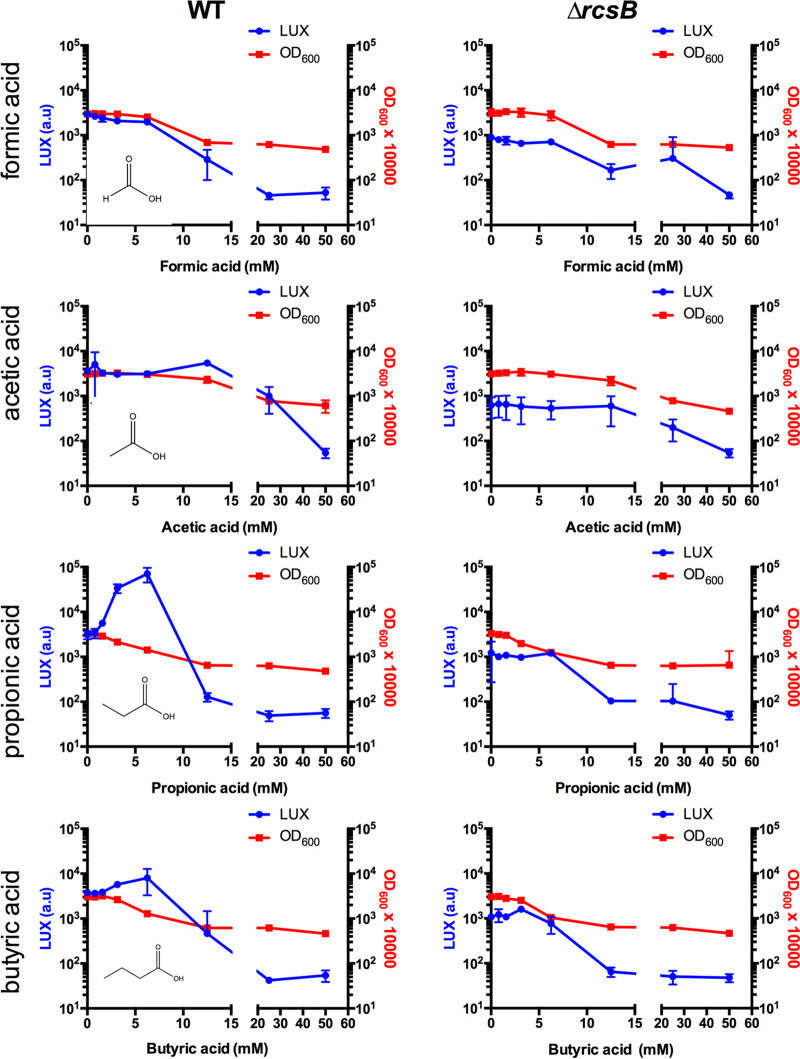
The short-chain fatty acid propionic acid activates the Rcs system. The S. marcescens wild-type and an isogenic Δ*rcsB* mutant were evaluated for P*_osmB_*-driven luminescence in the presence of short-chain fatty acids after coincubation for 4 h. The optical density at 600 nm was also determined. Propionic acid and butyric acid induced luminescence at subinhibitory concentrations in the wild-type but not in the Δ*rcsB* mutant. The mean and standard deviation values are shown. *n* ≥ 5.

SCFAs were chosen for further analysis, based on the data from the screen. These SCFAs were prioritized due to their high prevalence in the human gut and bloodstream as well as the lack of information regarding their impact on the Rcs system. The *P_osmB_-luxCADBE* construct was used to test four different SCFAs (Fig. 2). Formic acid and acetic acid had little impact on the reporter, whereas butyric acid and propionic acid strongly activated expression at subinhibitory SCFA levels. The highest activation was at a SCFA concentration of 6.25 mM. Notably, activation was largely or entirely absent in the Δ*rcsB* mutant, indicating a role for the Rcs system in this process. Similarly, the reporter was not induced by propionic acid in an *rcsC* mutant (Fig. S3), further implicating the Rcs system, rather than the alternative hypothesis that propionic acid leads to an increase in the acetyl-phosphate level, which has also been shown to activate RcsB (37).

10.1128/msphere.00439-22.3 FIG S3 Propionic acid induction of P*osmB* requires RcsC. The S. marcescens wild-type and an isogenic *rcsC* mutant were evaluated for P*osmB*-driven luminescence in the presence of short-chain fatty acids after coincubation for 4 h. The optical density at 600 nm was also determined. Propionic acid increased the luminescence in the WT but not in the *rcsC* mutant. The mean and standard deviation values are shown. *n* ≥ 10. Download FIG S3, TIF file, 0.2 MB. Copyright © 2023 Harshaw et al. 2023 Harshaw et al. https://creativecommons.org/licenses/by/4.0/ This content is distributed under the terms of the Creative Commons Attribution 4.0 International license.

**FIG 3:**
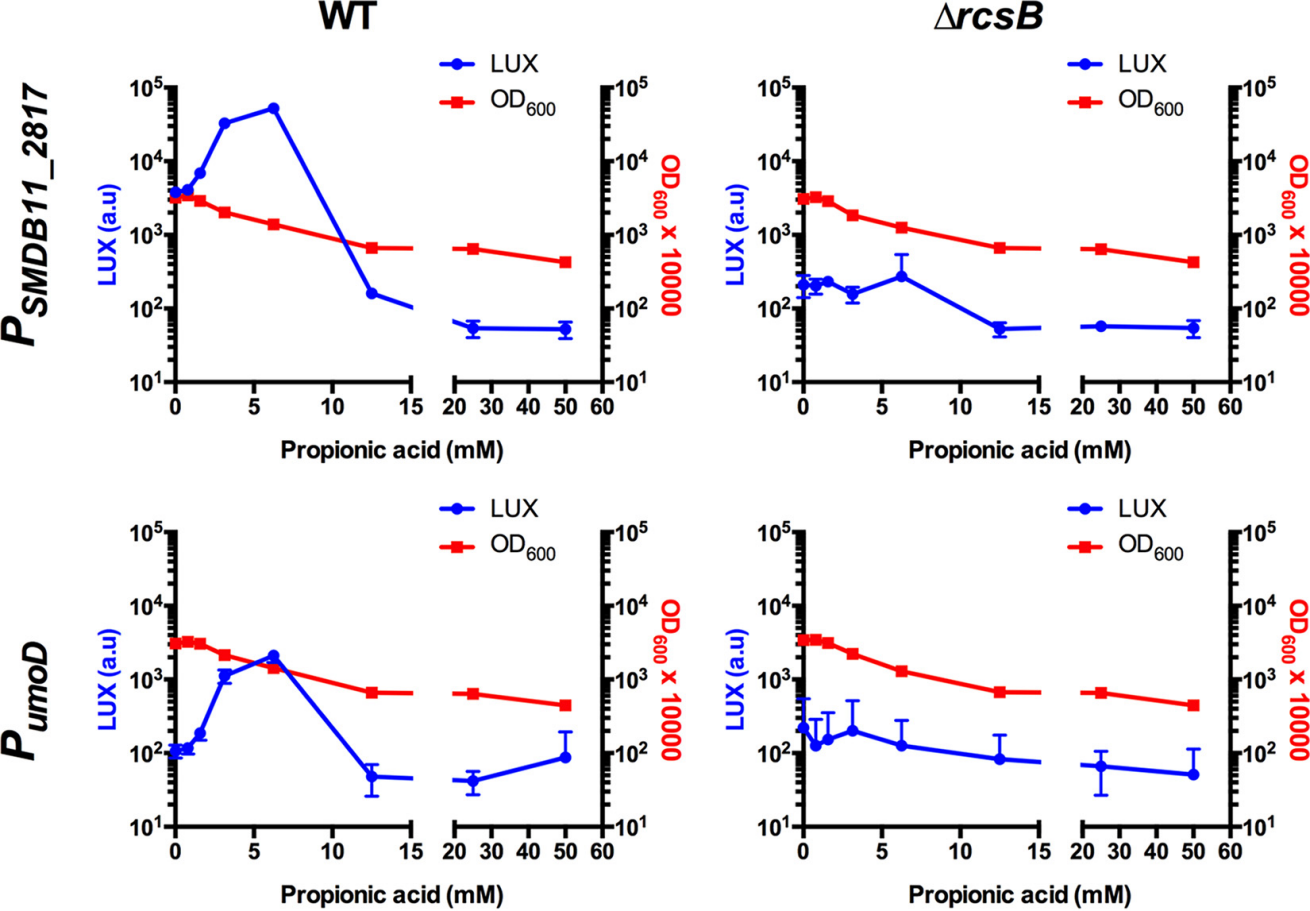
Propionic acid activates the Rcs-dependent promoters P*_SMDB11_2817_* and P*_umoD_*. S. marcescens strains with two different RcsB-influenced promoters driving *luxCDABE* produced increased luminescence in the presence of propionic acid after coincubation for 4 h. The optical density at 600 nm was also determined. The mean and standard deviation values are shown. *n* ≥ 6. This indicates that the Rcs-dependent effect of propionic acid is common to multiple Rcs activated promoters.

To test whether this effect was common to other Rcs-regulated promoters, we selected two that were recently shown to activate Rcs in this strain (29). These promoters, namely, P*_SMDB11_2817_* and P*_umoD_*, are also highly responsive to propionic acid in the WT background but are minimally or not activated in the Δ*rcsB* mutant (Fig. 3).

### Propionic acid mechanism of growth inhibition and Rcs activation.

The previously hypothesized mechanism by which propionic acid inhibits bacterial growth has been through a reduction in the intracellular pH (38). Undissociated forms of organic acids readily penetrate into bacterial cells, where they dissociate, thus impeding pH homeostasis (39). To test whether propionic acid concentrations that induce the Rcs system in S. marcescens caused a reduction in the intracellular pH, we used the pH sensitive GFP variant pHLuorin2 (40). The construct was validated to produce fluorescence in a pH-dependent manner (R^2^ = 0.943) and was responsive to culture acidification through growth with glucose (2% wt/vol). Growth in medium supplemented with high levels of glucose reduced the intracellular pH from 7.1 in LB medium to 6.5 in LB with glucose (Fig. S5), which is below 6.8, which is the reported intracellular pH that inhibits bacterial growth (41).

10.1128/msphere.00439-22.5 FIG S5 Propionic acid does not activate the Rcs system through lowering intracellular pH. The pH sensitive GFP variant pHIuorin2 that was codon optimized for S. marcescens was used to evaluate the intracellular pH. Fluorescence was measured after overnight growth in LB broth in the presence and absence of propionic acid. *n* = 4. The mean and standard deviation values are shown. (A) A linear standard curve was obtained. (B) Wild-type grown in LB medium with 2% glucose (v/v) had reduced intracellular pH. The pH values of overnight cultures are given. Download FIG S5, TIF file, 0.2 MB. Copyright © 2023 Harshaw et al. 2023 Harshaw et al. https://creativecommons.org/licenses/by/4.0/ This content is distributed under the terms of the Creative Commons Attribution 4.0 International license.

**FIG 4:**
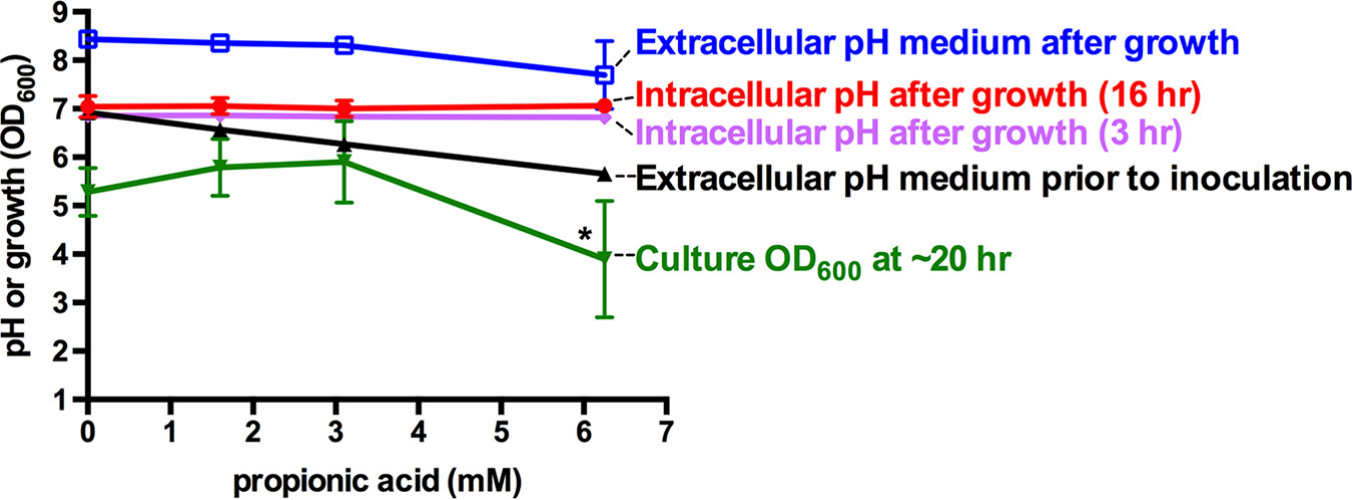
Propionic acid does not activate the Rcs system by lowering intracellular pH. The pH-sensitive GFP variant pHIuorin2 was codon optimized for S. marcescens and used to assess the intracellular pH. Fluorescence was measured from bacteria grown for 3 or 16 h in the presence of propionic acid. Propionic acid at concentrations that activate the Rcs system did not alter the intracellular pH at 3 h (pink diamonds) or 16 h (red circles). Prior to the bacterial inoculation, the pH of the medium was reduced with increasing propionic acid concentration (black triangles). After growth, the pH of the medium was made alkaline by bacterial growth effects (blue squares). The culture density was reduced when the bacteria were grown with 6.25 mM propionic acid but not with lower concentrations (green inverted triangles). Asterisks indicate statistically significant differences from 0 mM propionic acid (analysis of variance [ANOVA], Tukey’s *post hoc* test, *P* < 0.05). *n* = 4. The mean and standard deviation values are shown.

Increasing the propionic acid in the medium prior to inoculation resulted in a corresponding drop in the pH of the LB media, which decreased from 6.9 with no propionic acid to 5.7 with 6.25 mM propionic acid (Fig. 4). In the absence of propionic acid, the growth medium pH increased above 8, as was expected for S. marcescens (42, 43). In tubes with a propionic acid concentration of 6.25 mM, the pH lowered to 7.7 (Fig. 4). The intracellular pH was calculated to be between 7.0 and 7.1, regardless of the propionic acid concentration, at both early (3 h) and late (16 h) time points.

### Activation of Rcs through the inhibition of alanine racemase.

**FIG 5:**
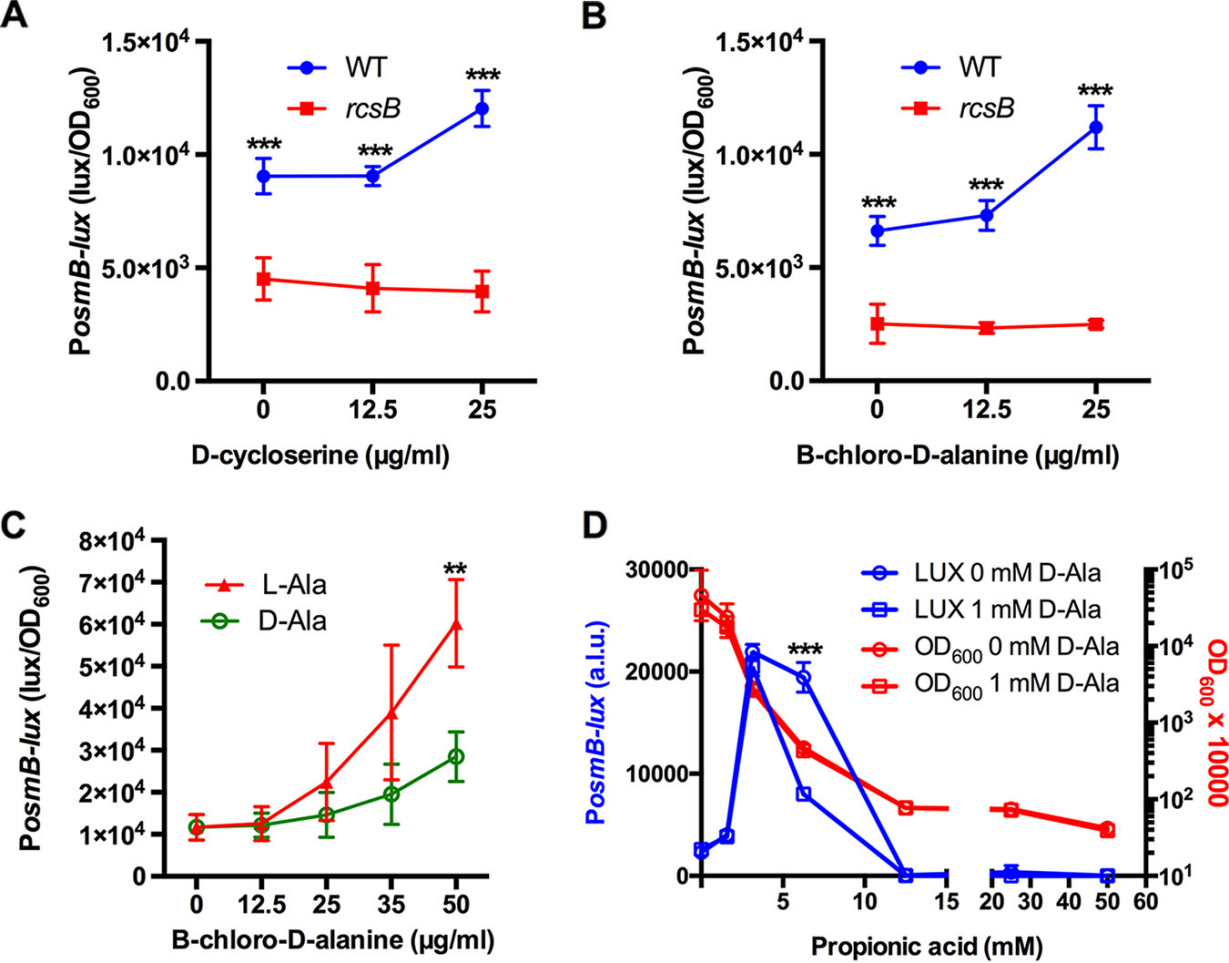
Alanine racemase inhibitors activate the Rcs system. Bacteria were grown in LB media with d-alanine racemase inhibitors for 4 h at 30°C. The P*_osmB_*-driven luminescence values were normalized by the culture density at 4 h postinoculation. (A) Luminescence response to d-cycloserine. (B) Luminescence response to β-chloro-d-alanine hydrochloride. (C) d-alanine but not l-alanine (1 mM) reduced the induction of *osmB* by β-chloro-d-alanine in the WT. (D) d-alanine reduced the luminescence activation by propionic acid. The mean and standard deviation values are shown. *n* ≥ 4. **, *P* < 0.001; ***, *P* < 0.0001.

These data suggest that it is unlikely that propionic acid activates the Rcs system through the acidification of the intracellular environments of bacterial cells. Propionic acid is a known inhibitor of alanine racemase for Bacillus stearothermophilus (44, 45), Staphylococcus aureus (46), and Streptomyces coelicolor (46). Alanine racemase is an essential enzyme for bacterial cell wall biosynthesis through the conversion of l-alanine to d-alanine, which is incorporated into the peptidoglycan cell wall (44, 47). The inactivation of alanine racemase prevents cell wall biosynthesis, which would be expected to activate the Rcs system and lead to bacterial cell death. If this is true, then alanine racemase inhibitors should activate the Rcs system. d-cycloserine is an inhibitor of both alanine racemase and d-alanine ligase. The P*_osmB_*-*lux* reporter was activated (>30%) in the WT by d-cycloserine at the maximum tolerated concentration of 25 mg/mL (245 μM), compared to no d-cycloserine (Fig. 5A). In contrast, the reporter was not activated in the Δ*rcsB* mutant (Fig. 5A). Another d-alanine racemase inhibitor, namely, β-chloro-d-alanine, was used, and it induced a stronger increase (69%) in expression from the P*_osmB_*-*luxCDABE* reporter at 25 μg/mL (156 μM) in the WT but not in the Δ*rcsB* mutant (Fig. 5B).

If the Rcs-dependent increase in the *P_osmB_*-*lux* reporter by propionic acid is triggered by the inhibition of the d-alanine racemase enzyme, then exogenous d-alanine would be expected to reduce cellular stress and correspond with lower luminescence values. To test this, bacteria were subjected to a range of β-chloro-d-alanine from 0 to 50 μg/mL in both the presence and absence of d-alanine or l-alanine (1 mM) (Fig. 5C). Similarly, d-alanine reduced the *P_osmB_* reporter activity in the 6.25 mM propionic acid challenge (Fig. 5D). However, consistent with propionic acid affecting cells in multiple ways, d-alanine did not rescue the growth inhibition by propionic acid. Together, these data suggest that propionic acid activates the Rcs system partially through the inhibition of the d-alanine racemase enzyme and that the inhibition of growth likely involves multiple targets for propionic acid and is not solely due to the inactivation of d-alanine racemase.

### Inhibition of S. marcescens alanine racemase activity by propionic acid *in vitro*.

**FIG 6:**
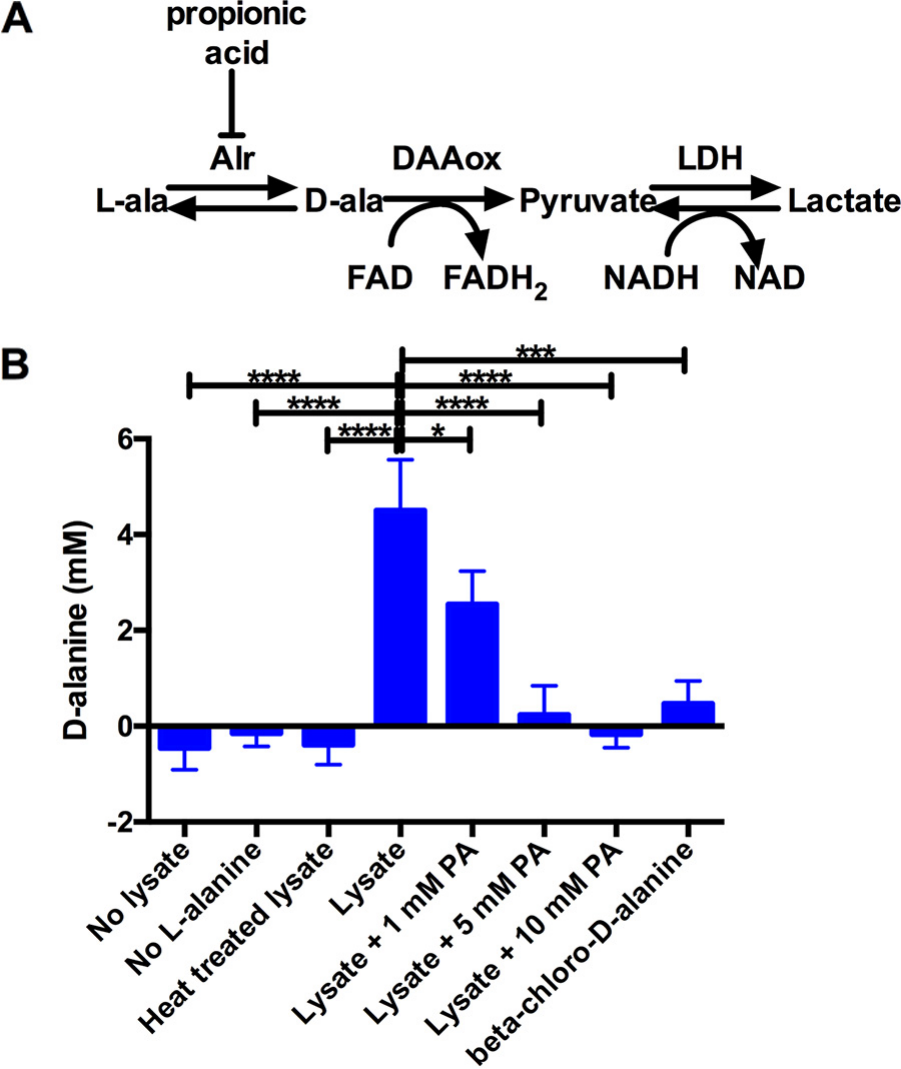
Propionic acid (PA) inhibits S. marcescens alanine racemase activity *in vitro*. (A) The schematic for the enzymatic activity for the conversion of l-alanine (l-ala) that was used in this assay. The alanine racemase activity (Alr) was provided from WT S. marcescens lysates. The reaction was measured by the change in NADH levels, as measured by the absorbance at 340 nm. DAAox is a d-amino acid oxidase. LDH is lactate dehyrogenase. (B) Indirect assay for the d-alanine concentration, based on the NADH oxidation, serves as an indicator for alanine racemase activity and for this activity as present in the lysate, but reduced by propionic acid or by the alanine racemase inhibitor β-chloro-d-alanine. l-alanine at 10 mM was introduced into the experiment, and its conversion to d-alanine was determined via a comparison against a standard curve. Statistical significance was determined using an analysis of variance (ANOVA) with Tukey’s *post hoc* test. *, *P* < 0.05; ****, *P* < 0.0001. The mean and standard deviation values are shown. *n* = 3.

A prerequisite for our model is that propionic acid can inhibit the S. marcescens alanine racemase. Whereas the inhibition of alanine racemase has been demonstrated with certain Gram-positive bacteria, it has not been demonstrated with Gram-negative bacteria, such as S. marcescens. An enzymatic approach was used to ascertain the alanine racemase activity in partially purified wild-type lysates from which compounds smaller than 10 kDa, such as d-alanine, were removed (see Materials and Methods) (Fig. 6A). The experiments indicated that the S. marcescens lysates contained robust alanine racemase activity (Fig. 6B).

To evaluate whether propionic acid inhibited S. marcescens alanine racemase activity *in vitro*, propionic acid was added to the lysate and was found to inhibit alanine racemase in a dose-dependent manner (Fig. 6B). Similarly, the alanine racemase inhibitor β-chloro-d-alanine inhibited the reaction as expected (Fig. 6B). As a control, it was determined that propionic acid did not inhibit other enzymes required for the analysis, indicating that the inhibitory effect was on alanine racemase (Fig. S6). Together, these data suggest that S. marcescens alanine racemase activity can be inhibited by propionic acid and support the model that propionic acid activates the Rcs system partly through the inhibition of alanine racemase activity.

10.1128/msphere.00439-22.6 FIG S6 Alanine racemase assay additional controls. Controls for the enzymatic alanine racemase assay (Fig. 6A). The complete experiment with no added bacterial lysate did not give a measurable background signal (column 1). The complete assay without added l-alanine did not provide a signal, indicating that there were no detectable l-alanine or d-amino acids in the bacterial lysate (column 2). The complete assay without the d-amino acid oxidase (DAAox) did not provide a positive signal, indicating that the bacterial lysate did not lead to a false positive signal, compared to the standards (column 3). The second and third enzymatic reactions were not inhibited by propionic acid (PA), indicating that the effect of propionic acid on the lysate was upon alanine racemase in the lysate (column 4). The mean and standard deviation values are shown. *n* = 3. Download FIG S6, TIF file, 0.1 MB. Copyright © 2023 Harshaw et al. 2023 Harshaw et al. https://creativecommons.org/licenses/by/4.0/ This content is distributed under the terms of the Creative Commons Attribution 4.0 International license.

### Propionic acid impacts flagella-based motility and *fliC* gene expression.

**FIG 7:**
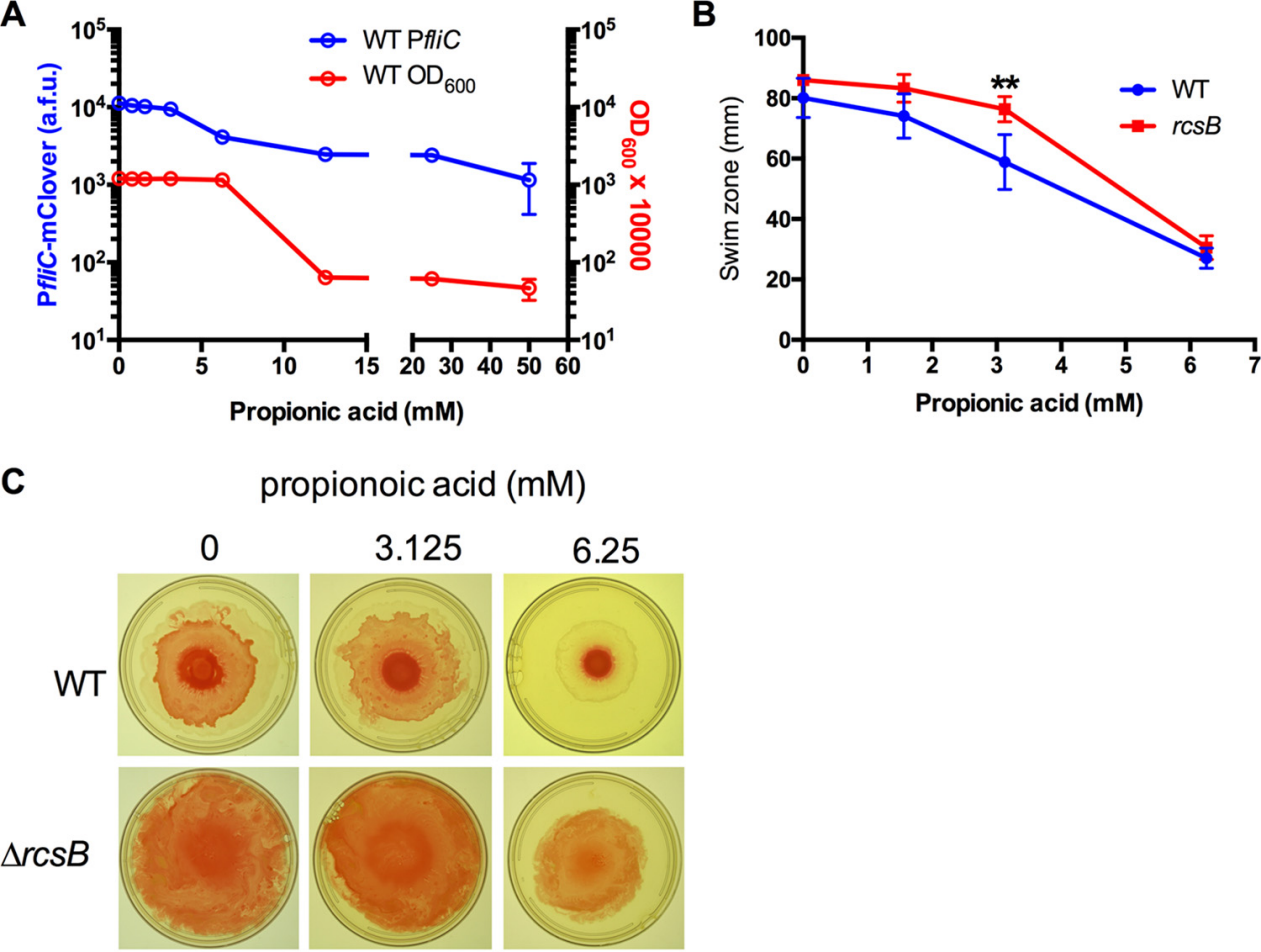
Regulation of *fliC* and flagella-based motility by propionic acid. (A) Fluorescence from the *fliC* transcriptional reporter and the corresponding optical density after 16 h at 30°C. *n* = 16. (B) Swim zone at 16 h at 30°C in LB with agar at 0.3% (wt/vol). **, *P* < 0.01. *n* ≥ 5. (C) Representative images of swarming motility on LB agar (1% agar).

The bacterial flagellum is a potent activator of inflammation through binding with TLR5 (48). Flagellar genes are regulated by the Rcs system in numerous species, including S. marcescens (19, 26). We tested whether physiologic concentrations of propionic acid had an impact on S. marcescens flagellum-based motility (Fig. 7). A *fliC* promoter-mClover transcriptional reporter demonstrated increased fluorescence over time in the WT. So, an overnight (16 h) culture was used to evaluate the effect of propionic acid. For the WT, increasing propionic acid levels correlated with reduced fluorescence, even when growth was largely unaffected (Fig. 7A). The Δ*rcsB* mutant was not used because it has the *rcsB* gene replaced by *mClover*, and we could not distinguish between P*fliC*-mediated and P*rcsB*-mediated mClover fluorescence. Swimming motility was evaluated to determine whether this change in *fliC* expression in the WT conferred a phenotype (Fig. 7B). Both the WT and Δ*rcsB* mutant were inhibited for swimming zones, with a greater effect being observed on the WT (Fig. 7B). As expected, swarming motility was distinctly greater in the Δ*rcsB* mutant due to derepressed flagellar operons (49–51), and both genotypes had swarming partially inhibited by propionic acid at 6.25 mM (Fig. 7C). These data suggest that propionic acid can inhibit flagellar based motility, both by Rcs-dependent and independent mechanisms.

### D-alanine dependent activation of an Rcs-reporter in an E. coli *alr dadX* mutant.

**FIG 8:**
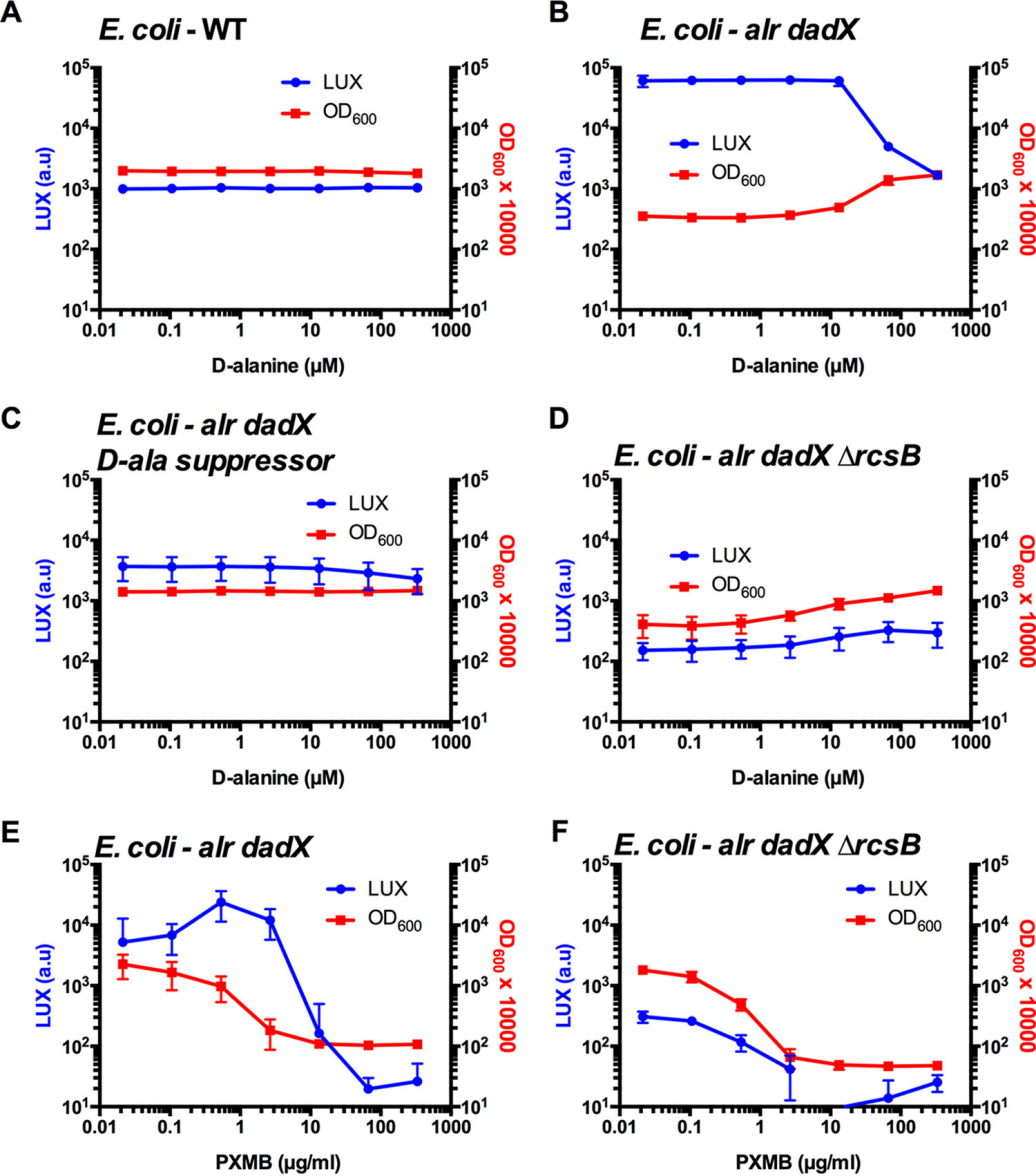
Increased promoter activity in the absence of alanine racemase activity in E. coli. The E. coli wild-type (A) and an isogenic *alr dadX* mutant (d-alanine auxotroph) (B) were evaluated for P*_SMDB11_2817_*-driven luminescence in the presence and abscence of d-alanine in LB medium after coincubation for 4 h. The same promoter was evaluated in a variant of the *alr dadX* suppressor mutant that was able to grow without d-alanine (C) and in a *alr dadX* Δ*rcsB* triple mutant (D) at different concentrations of d-alanine. The optical density at 600 nm was also determined. The results suggest that the Rcs system is activated in the absence of alanine racemase in E. coli and verify that the promoter is Rcs-regulated in E. coli. (E and F) The known Rcs activator polymyxin B (PMXB) induced P*_SMDB11_2817_*-driven luminescence in the *alr dadX* mutant but not in the *alr dadX* Δ*rcsB* mutant when grown in LB with d-alanine (2 mM). The mean and standard deviation values are shown. *n* = 12.

If the inhibition of alanine racemase activity activates the Rcs system, then an alanine racemase defective strain should increase Rcs-gene expression. In E. coli there are two independent alanine racemases that are coded by the *alr* and *dadX* genes, and double mutants are auxotrophic for d-alanine (52). We grew wild-type strain MG1655 (53) with pMQ748 (P*_SMDB11_2817_-luxCDABE*) in LB medium with different levels of d-alanine (Fig. 8A). No changes in luminescence or growth were observed. Importantly, this S. marcescens promoter appears to be Rcs regulated in E. coli, as it was strongly induced by subinhibitory concentrations of the known Rcs-inducing and membrane-targeting antibiotic polymyxin B (54), with a 16.2 ± 3.4-fold induction in luminescence for the WT that was exposed to 0.2 μg/mL polymyxin B, compared to the no antibiotic control at 4 h.

Strain MB2159 has Δ*alr* and Δ*dadX* mutations and cannot grow in LB broth without supplemental d-alanine (52). MB2159 with pMQ748 demonstrated d-alanine-dependent growth, and luminescence increased at growth-limiting d-alanine concentrations (Fig. 8B).

We isolated a spontaneous d-alanine prototroph of strain MB2159 that was able to grow on LB media without d-alanine (Fig. 8C). These can occur in Δ*alr* Δ*dadX* strains that experience mutations that upregulate the expression of *metC*, which codes for a cystathionine β-lyase that can provide d-alanine (55). This prototrophic variant was able to grow without d-alanine, and, with pMQ748, its luminescence was minorly or not altered with reduced d-alanine (Fig. 8C). This directly links d-alanine auxotrophy, the lack of alanine racemase activity, with the increased expression of an Rcs-reporter construct.

To further demonstrate the necessity of the Rcs system with these increased reporter levels, we generated a Δ*alr* Δ*dadX* Δ*rcsB* triple mutant and found that while it was still dependent on d-alanine for growth, it did not have increased luminescence in response to d-alanine concentrations (Fig. 8D). Together, these data support the model that in E. coli, the loss of alanine racemase activity leads to the activation of the Rcs system.

The Δ*alr* Δ*dadX*, but not the Δ*alr* Δ*dadX* Δ*rcsB* strain, exhibited polymyxin B-dependent induction of the P*_SMDB11_2817-_luxCDABE* reporter (Fig. 8E and F). The results of this control experiment further support the Rcs-specificity of the reporter in E. coli.

## DISCUSSION

Our screen for compounds that induce an Rcs system-regulated promoter has identified new activators and has led us to evaluate the short-chain fatty acid propionic acid. In some cases, Rcs-activated promoters can be controlled by other stress response systems (7). Strong support for propionic acid activating the Rcs system is its ability to activate three different Rcs-regulated promoters in the WT but not in an isogenic Rcs-deficient mutant. Due to its antimicrobial effects, propionic acid is commonly used as a food preservative (39) and topically to treat skin infections (56), and it may influence the susceptibility of bacteria to other antibiotics (57, 58). However, the antimicrobial mechanism of action is not fully understood, and it has been hypothesized to be derived from intracellular acidification (39). At this point, it is not clear whether intracellular acidification would strongly or directly activate the Rcs system, and indeed, we saw that propionic acid did not cause intracellular acidification at concentrations that activated the Rcs system reporter. However, the protonation of the lipopolysaccharides (LPS) of S. enterica grown in acidic media has been demonstrated to activate Rcs as well as PhoPQ-based signaling (59). So, external pH may contribute to the observed propionic acid effect. Due to these observations, we tested an alternative hypothesis that propionic acid inhibits d-alanine racemase activity, as was shown for several Gram-positive bacterial species. The inhibition of this key enzyme in cell wall biosynthesis would be expected to activate the Rcs system. Our data suggest that subinhibitory concentrations of known d-alanine racemase inhibitors increase expression from an Rcs dependent promoter in an RcsB-dependent manner and that exogenous d-alanine can quell this induction. Furthermore, our data support the propionic acid inhibition of S. marcescens d-alanine racemase *in vitro*.

Similarly, in E. coli, the loss of alanine racemase due to *alr* and *dadX* deletion mutations correlated with increased Rcs-reporter expression when the mutant was grown at growth-limiting d-alanine concentrations. Strengthening the link between alanine racemase activity and Rcs-activation is a suppressor mutation that restored alanine racemase function to the alanine racemase mutant (eliminated the growth requirement for exogenous d-alanine) and thereby restored the WT phenotype. That is, the Rcs reporter became indifferent to d-alanine levels. Similarly, the alanine racemase defective mutant, in which the Rcs system was activated through the mutation of *rcsB*, also lost Rcs reporter activation, despite remaining auxotrophic for d-alanine. Together, these data support a role for d-alanine racemase activity being sensed by the Rcs system and further indicate that the effect is not specific to S. marcescens.

However, in S. marcescens, promoter activation by d-alanine inhibitors did not achieve that of propionic acid, suggesting that propionic acid has additional mechanisms of Rcs system activation. For instance, the bacterial outer membrane lipid content and protein profiles can be influenced by short-chain fatty acids, such as propionic acid, in Borrelia burgdorferi, Prevotella bryantii, and Pseudomonas aeruginosa (58, 60, 61). Freese, et al. hypothesized that the antimicrobial target of short-chain fatty acids is the membrane (62). Additionally, cell surface, pH-dependent LPS protonation may contribute to this effect (59). These membrane effects may also contribute to the propionic acid induction of the Rcs system.

SCFAs, such as butryrate and propionic acid, impact the immune system and can influence host responses to herpes simplex virus (63) as well as the outcomes of dry eye diseases (64). They can impact host responses by binding to the FFAR2 G-protein-coupled receptor and influencing the response to LPS (65, 66). The bacterial behaviors of Gram-negativity and Gram-positivity are also highly influenced by SCFA (67, 68). Our study showed a measurable, if modest, impact of propionic acid on flagella-based motility. As the flagellum is a major pathogen-associated molecular pattern, this inhibition could influence host-pathogen interactions.

Our study also confirmed several established Rcs activating compounds and compound classes, such as β-lactam antibiotics. General membrane-targeting antimicrobial compounds, such as polymyxin B, were also identified. Here, we find three previously unreported activators of the Rcs system: chelerythrine, cinnamic acid, and 4-aminopyridine, which are molecules that were predicted to be a transporter inhibitor, a permeability changer, and a proton motive force uncoupler, respectively. Other predicted PMF uncouplers, namely, ethanol and antimicrobial peptide Gramicidin A, are known to activate the Rcs system (6). We also observed that the proton motive force uncoupler carbonyl cyanide *m*-chlorophenyl hydrazone increased reporter activity by approximately eightfold above that of the Δ*rcsB* mutant, but this did not reach our threshold of significance (*P* = 0.12). SCFAs may also affect membranes and membrane protein functions through the disruption of the proton gradient (39).

Questions remain regarding how physical damage activates the Rcs system. Whereas outer membrane perturbations can activate the system, this is not always the case. A systematic analysis by Steenhuis and colleagues demonstrated that compounds that either increased outer membrane permeability (edthylenediaminetetraacetic acid [EDTA] and sodium dodecyl sulfate [SDS]) or compromised outer membrane integrity (triclosan) failed to activate the Rcs system of E. coli (7). The authors concluded that the Rcs system responds to the interaction of antimicrobial peptides with LPS, rather than the permeabilization of the outer membrane, and this is likely due to the RcsF monitoring of the LPS layer (7). Nevertheless, the extent of Rcs activation by peptides was variable, even at 0.5× MIC values, and the difference could not be explained simply by the molecular weights of the tested compounds (7).

The impact of cell wall acting antibiotics on the induction of the Rcs system is also similarly nuanced. A number of β-lactams in this study strongly activated the Rcs system, whereas others did not. This may not just be an issue of susceptibility, as the S. marcescens Rcs system can be activated by the cell wall targeting antibiotics to which it is resistant, such as cefazolin and vancomycin (29). In a study by Sailer et al., numerous peptidoglycan antibiotics were tested for their activation of the *cps* genes, a classic output for the activation of the Rcs system in E. coli. Interestingly, despite inhibiting bacterial growth, approximately half of the tested β-lactams were unable to activate the expression of a *cps* transcription (9). This was not obviously due to the inhibition of specific penicillin binding proteins (PBPs), although it is clear that the simultaneous mutation of several PBPs (PBP4, PBP5, PBP7, and AmpH) is sufficient to activate the Rcs system (69). Other differences between studies have arisen. For example, ampicillin causes the induction of a fluorescent reporter for the Rcs regulated *rprA* gene (7) and increases the expression of Rcs controlled genes, as determined by a microarray in E. coli (10). In contrast, in our study, ampicillin induced a modest 30% induction of an Rcs-dependent promoter in the WT, compared to the Δ*rcsB* mutant. Some differences in study outcomes may be due to the differences in the sensitivity of the assay, the time point of the analysis, the concentration of the drug tested, and the differences between species with respect to drug permeability. Alternatively, the different effects may indicate that highly specific, rather than general, insults are detected by the Rcs system.

In conclusion, this study identified the SCFA propionic acid as a strong inducer of the Rcs stress response system of S. marcescens. Due to the important role of the Rcs system in controlling bacterial behaviors, such as virulence factor production, this suggests a role for SCFAs in the gut in programming enteric bacteria to reduce virulence factor production. The Rcs system is, in general, highly conserved among the tested Enterobacterales, with notable exceptions, such as Yersinia pestis, which has a highly active RcsC, thereby highlighting the need for validation in other Gram-negative species (70). This study also underscores the potential of SCFAs as alternatives or adjuvants to antibiotics that may also reduce virulence factor production.

## MATERIALS AND METHODS

### Reporter assays.

Glycerol stocks of the S. marcescens strains were maintained at −80°C and are listed in Table 1. All incubations of S. marcescens were performed between 30 and 32°C in lysogeny broth (LB) with or without agar. The LB medium was supplemented with 10 μg/mL gentamicin to maintain only cells that contained the luminescence reporter plasmid. To obtain single colonies, S. marcescens strains were grown overnight on LB agar, streaking for isolation. Single colonies were then grown overnight in LB with gentamicin, receiving aeration on a tissue culture roller. After approximately 18 h, the overnight cultures were normalized in LB with gentamicin to OD_600_ = 0.1. 100 μL of normalized culture were added to each well of Biolog GenIII and Phenotype MicroArray 11 to 20 MicroPlates. The plates were rocked for 5 min to ensure the proper dissolution of the plate compounds. The plates were placed in a plastic bag with a wet paper towel and incubated between 30 and 32°C. The wells were transferred to black-sided, clear-bottomed, 96-well plates (Nunc 165305) to prevent luminescence cross-contamination from one well to another. The Lux and OD_600_ readings were taken after 4 h, and, in some cases 6 h, of incubation time. The relative Lux values were calculated by dividing the Lux value by the OD_600_. These values were then normalized to the control well to determine the fold change in Lux expression that was caused by each plate compound.

*mClover* reporter assays were performed as described above, except that the fluorescence was measured from the bacteria in black-sided, clear-bottomed, 96-well plates (Nunc 165305) with a plate reader (Biotek Synergy 2), using 485/20 nm excitation and 516/20 nm emission filters. The optical density at 600 nm was also measured. The fluorescence was measured after overnight growth (approximately 18 h).

### Molecular biology and intracellular pH analysis.

The *rcsB* gene was replaced with a kanamycin resistance cassette from the plasmid pKD4 (71) using lambda red recombineering that was enabled by plasmid pMQ538, as previously described (71, 72). The kanamycin resistance cassette was amplified with oligonucleotide primers that amplify the cassette and have 40 bp tails to target recombination on the E. coli chromosome (5′ to 3′: agttatgtcaagagcttgctgtagcaaggtagcctattacGTGTAGGCTGGAGCTGCTTC and ataagacactaacgcgtcttatctggcctacaggtgaGGTCCATATGAATATCCTCCTTA, with the upper case letters directing amplification). The resulting mutant strain was verified by polymerase chain reaction (PCR) with flanking and internal primers that verified both junctions.

The pH-sensitive *gfp* variant pHluorin2 (40) was codon optimized for S. marcescens using online software (Integrated DNA Technologies), and it was placed under the control of the constitutive *nptII* promoter. A synthetic DNA fragment with the pHluorin2 open reading frame was designed to recombine with the fluorescent protein expression vector pMQ414 (73) using yeast *in vivo* cloning (74). The sequence from 5′ to 3′ is ggcgtttcacttctgagttcggcatggggtcaggtgggaccaccgcgctactgccgccaggcaaattctgttttatcagaccgcttctgcgttctgatttaatctgtatcaggatccTTTATACAGTTCGTCCATGCCGTGGGTTATGCCCGCAGCAGTCACAAACTCGAGCAGCACCATGTGGTCCCGCTTTTCATTGGGGTCTTTTGAGAGGGCCGATTGGGTGTGGAGGTAGTGGTTGTCTGGCAACAGTACTGGACCATCCCCAATTGGAGTGTTTTGCTGATAATGGTCGGCAAGCTGTACGCTGCCATCTTCAATATTATGGTGCACTTGAAAAATGGCCTTTGTTCCATTTTTCTGCTTGTCCGCCATGATATACACGAGATGTTCATTATAGTTGTACTCGAGTTTATGCCCCAGTATGTTACCGTCTTCCTTAAAGTCTATCCCTTTGAGTTCTATCCGATTTACCAACGTGTCCCCCTCGAACTTTACCTCGGCGCGTGTCTTGTAGTTCCCGTCATCCTTGAAGAATATAGTCCGCTCCTGGACATATCCTTCGGGCATTGCAGACTTGAAGAAGTCATGCTGCTTCATATGATCGGGGTAGCGGGAAAAACACTGGACTCCGTACGACAGTGTTGTGACGAGCGTGGGCCACGGAACTGGCAATTTTCCGGTCGTGCATATAAATTTGAGGGTCAACTTACCATATGTTGCGTCACCCTCTCCTTCTCCCGAAACCGAAAATTTGTGCCCGTTAACATCACCGTCCAATTCGACGAGTATAGGGACCACACCTGTGAACAATTCCTCGCCTTTGCTCATgaattctcctcatcctgtctcttgatcagatcttgatcccctgcgccatcagatccttggcggcaagaaagccatccagtttactttgcagggcttcccaaccttaccagagg. The pMQ414 vector was cut with BamHI and EcoRI to remove *tdtomato*, and it was moved into the Saccharomyces cerevisiae strain InvSc1 with the synthetic DNA, as previously described (74). The resulting plasmid pMQ802 was verified by PCR, and the junctions were sequenced. The plasmid was moved into S. marcescens strain K904 via conjugation.

The intracellular pH was determined as previously described (40). First, a standard curve was established using buffered medium. Cultures of S. marcescens with plasmid pMQ802 were grown in 5 mL LB for 16 h at 30°C with aeration and were either taken for analysis or subcultured 1:50 and grown for 3 h before sampling. 1 mL aliquots of normalized subcultures (OD_600_ = 2) were decanted into microcentrifuge tubes and pelleted by centrifugation. The pellets were resuspended in 1 mL of buffers (50 mM) at pH 5.5 MES (Sigma, product M-5057), 7.0 MOPS (Sigma, product M1254), and 8.3 TAPS (Sigma, product T5130), along with 50 mM methylamine HCl (Sigma, product M0505) and 50 mM sodium benzoate (ThermoFisher, Heysham, Lancashire, LA3 2XY, UK, product A15946). 0.2 mL aliquots were transferred into the wells of a black-sided, clear-bottomed, 96-well plate (ThermoFisher, Waltham, MA, USA, product 165305). The OD_600_ and the fluorescence were recorded using a plate reader (Molecular Devices SpectraMax M3, San Jose, CA, USA). The fluorescence measurements were at excitation/emission wavelengths of 485/515 and 405/515. Then, the ratio of the fluorescence at excitation 405 over 485 (R_405/485_) was calculated for the construction of a calibration curve. These were compared to a buffer blank, and a linear standard curve was established.

To test the effect of propionic acid (various concentrations) and glucose (2% wt/vol) on the fluorescent activity of the pH dependent GFP, cultures of S. marcescens with plasmid pMQ802 were started from −80°C glycerol stocks with 5 mL LB, 5 μL gentamicin, and a specified concentration of PA or glucose. The concentrations of the PA supplemented growth media were 1.6 mM, 3.1 mM, and 6.3 mM (Sigma, product P1386). The effect of PA was measured with the same procedure used in the verification of the pH-dependence of S. marcescens with plasmid pMQ802. However, during the resuspension, the pellets were resuspended in 1 mL of a solution containing 50 mM methylamine HCl and 50 mM sodium benzoate. 200 μL aliquots from the resuspended cultures were organized into the wells of a black, 96-well clear-bottomed plate. The OD_600_ and the fluorescence were measured at 485/515 and 405/515, and the ratios were compared to the standard curve.

### Enzymatic assay.

Overnight cultures of wild-type bacteria were subcultured 1:100 (vol/vol), grown to OD_600_ = 1.65 in LB broth at 37°C in a 100 mL volume, spun down by centrifugation (7 min at 15,000 × *g*), washed with an equal volume of cold PBS, and frozen at −20°C. The pellets were suspended in PBS, sonicated to lyse the cells, and centrifuged (2 min at 15,000 × *g*). The lysate was further clarified by passing through a 0.22 μm filter and a 10 kDa size exclusion filter (Amicon, Millipore UFC901008), and it was washed with PBS to facilitate the removal of small molecules, such as d-alanine. The lysates were normalized to 23.3 mg/mL using PBS, following Bradford protein analysis. Glycerol was added to 5% and the partially purified lysates were stored at –20°C or 4°C until use. The experiments were performed three times with two independent lysate preparations, and the results were consistent with a pilot assay using a third independent lysate.

Closely following the protocol of Garrett et al. (75), the lysate was assessed for alanine racemase activity. The experiment was performed in three reactions, with each being stopped via incubation at 85°C for 10 min. In the first reaction, the lysate (50 μL) was mixed with l-alanine to a final volume of 10 mM in PBS in a total volume of 0.1 mL, and this was incubated at 37° for 1 h. In this step, any alanine racemase present in the lysate could convert l-alanine to d-alanine. The negative controls for this step included no l-alanine addition and the heat treatment of the lysate prior to the assay.

For the second assay, the entire first reaction was added to a tube containing d-amino acid oxidase (20 units/mL, 10 μL, Sigma A5222), catalase (40 units/μL, 10 μL, Sigma C9322), FAD (1 mg/mL, 5 μL, Sigma F8384), and PBS in a total volume of 1.705 mL. This reaction proceeded for 1 h at 37°C and was stopped via heating, as described above. Here, the d-amino acid oxidase converts d-alanine into pyruvate. A no d-amino acid oxidase control was included as a negative control.

The third step involved the addition of 1 mL of the reaction mixture with 1 mL PBS in a cuvette. NADH (10 mg/mL, 60 μL, Millipore 10128023001) was then added to all cuvettes, and the mixture was allowed to incubate for 3 to 5 min. The absorbance at 340 nm was measured across a 1 cm path length in a 1 cm path length cuvette. Following the first A_340_ reading, lactate dehydrogenase (7,200 units/mL, 10 μL, Millipore 427217) was added to all of the cuvettes. After 5 min of incubation, the A_340_ was read again. The change in absorbance before and after the addition of lactate dehydrogenase determined the conversion of NADH to NAD^+^. Cuvettes containing known millimolar quantities of d-alanine were added to the second reaction to obtain a standard curve for comparison against the reaction conditions, and it was linear from 0.1 to 100 mM d-alanine.

To test the inhibitors, the experiment was performed as described above, but propionic acid was added to the first reaction at 1, 5, and 10 mM. A known alanine racemase inhibitor, namely, β-chloro-d-alanine (0.625 mM, Sigma C4284), was used as a positive control for the inhibition. Propionic acid at 10 mM was also added to the second reaction with d-alanine (10 mM) to ensure that propionic acid did not inhibit either d-amino acid oxidase or lactate dehydrogenase.

### Literature search.

To identify whether compounds have been previously determined as activating the Rcs system, the following searches were taken: (i) Google Scholar “Rcs system” and “compound name”, (ii) Google Scholar “RcsB” and “compound name”, (iii) NCBI PubMed “Rcs system” and “compound name”, and (iv) NCBI PubMed “RcsB” and “compound name”. Compounds were listed as not previously reported if no specific description of the compound activating the transcription of Rcs-dependent genes was previously described, based on the literature search. These are listed in Table 3.

**TABLE 3:** Compounds that induce the *P_osmB_-luxCDABE* reporter

Compound	Plate	Compound class	Function	Fold WT compound/control^*a*^	Fold (WT/Δ*rcsB*)^*b*^	*P* value^*c*^
Phenethicillin	PM19	Beta-lactam	Cell wall targeting	86.0	74.9	0.001
Sodium azide	PM15	Inhibitor of oxidative phosphorylation	Metabolic compounds/effectors	34.0	22.7	0.006
Sodium caprylate	PM19	Medium-chain fatty acid	Other	19.4	12.9	0.000
Cinnamic acid	PM19	Cell membrane effector	Transport inhibitors/permeability change/PMF uncoupling	9.9	8.5	0.042
Polymyxin B	PM19	Polymyxin	Cell wall targeting	6.4	5.5	0.003
e,l-thioctic acid	PM19	Redox compound	Redox compound	5.3	5.2	0.007
Vancomycin	PM12	Glycopeptide antibiotic	Cell wall targeting	5.4	4.4	0.025
Menadione	PM15	Medication	Repurposed medication	3.7	4.1	0.003
1-hydroxy-pyridine-2-thione	PM14	Chelator	Chelating agents and surfactants	4.1	3.7	0.018
Chelerythine	PM14	Permeability effector	Transport inhibitors/permeability change/PMF uncoupling	4.8	3.5	0.038
Cefoxitin	PM14	Cephalosporin	Cell wall targeting	4.0	3.4	0.005
Promethazine	PM14	Medication/metabolic inhibitor	Repurposed medication	4.7	3.3	0.033
Sodium arsenate	PM14	General toxin	Other	2.1	2.9	0.032
Amoxicillin	PM11	β-lactam	Cell wall targeting	2.5	2.6	0.002
Sulfamethazine	PM12	Sulfonamide	Nucleic acid synthesis	3.6	2.6	0.007
Penimepicycline	PM12	Tetracycline	Protein synthesis	3.2	2.5	0.039
2-phenylphenol	PM18	Disinfectant	Disinfectants	2.9	2.4	0.012
Sodium m-periodate	PM18	Redox compound	Redox compound	2.5	2.4	0.006
Oxycarboxin	PM17	Inhibitor of oxidative phosphorylation	Metabolic compounds/effectors	2.2	2.2	0.027
Nafcillin	PM11	β-lactam	Cell wall targeting	2.3	2.2	0.001
Disulphiram	PM19	Medication	Repurposed medication	2.2	2.2	0.047
Carbenicillin	PM12	β-lactam	Cell wall targeting	2.2	2.1	0.004
Cloxacillin	PM11	β-lactam	Cell wall targeting	2.2	2.1	0.033
Iodoacetate	PM14	Protein synthesis inhibitor	Protein synthesis	1.9	2.0	0.020
Nitrofurantoin	PM14	Nucleic acid synthesis inhibitor	Nucleic acid synthesis	2.3	2.0	0.002

a Average of the WT levels with compound over the WT levels in the control wells. *n* = 3.

b Average of the WT levels divided by the Δ*rcsB* mutant levels. *n* = 3.

c Student’s *t* test of the WT levels versus the Δ*rcsB* levels.

### Statistical analysis.

The GraphPad Prism software package was used to perform analyses of variance (ANOVA) with Tukey’s *post hoc* test or Student’s *t* test.

### Data availability.

The authors will provide data upon request.
