# Supplementary figures and images for: The Short-chain Fatty Acid Propionic Acid Activates the Rcs Stress Response System Partially through Inhibition of d-Alanine Racemase
Source: mSphere. 2023 Jan 16;8(1):e00439-22. doi: 10.1128/msphere.00439-22 (PMC9942566; doi:10.1128/msphere.00439-22)

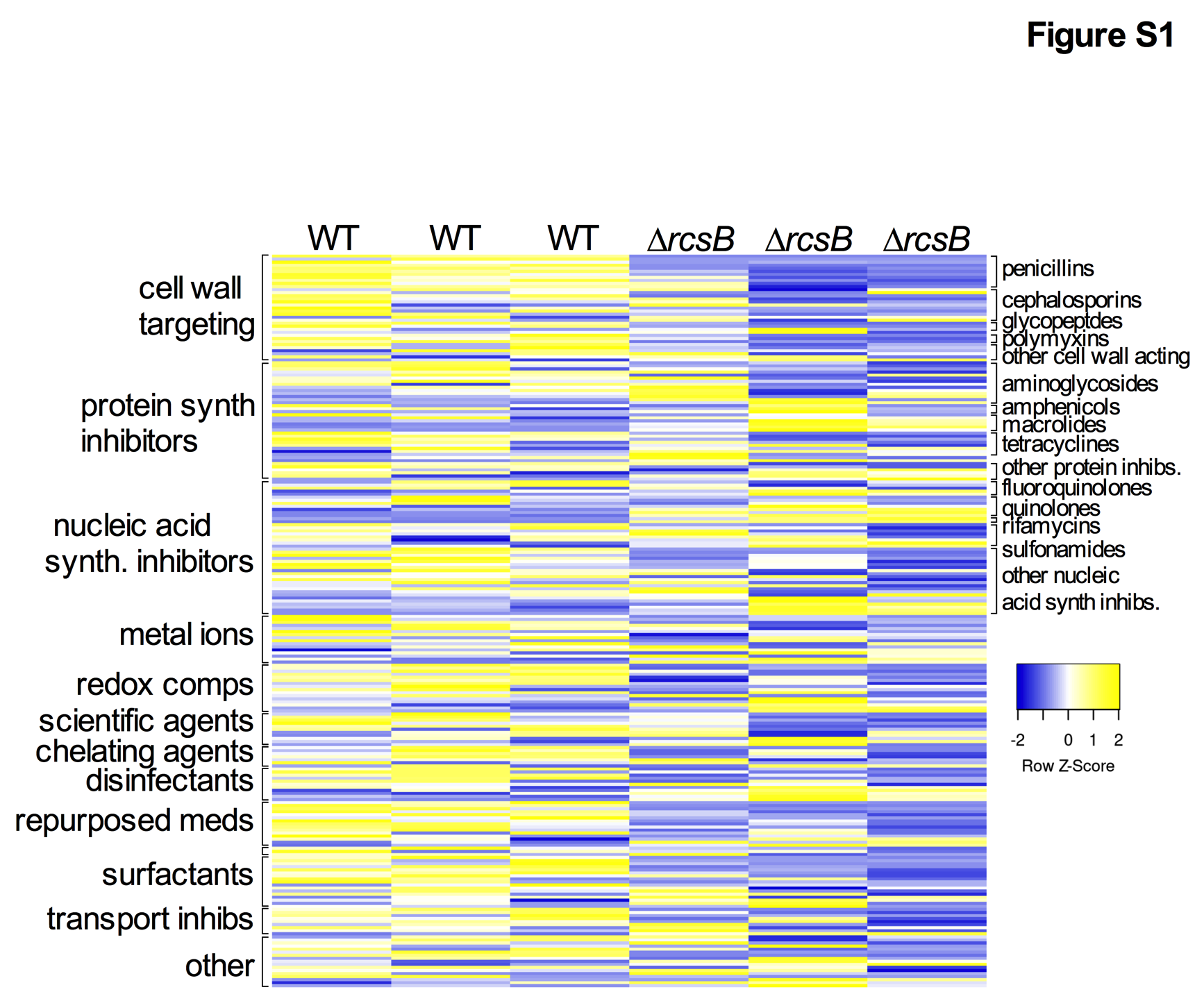

Supplement: FIG S1 [file msphere.00439-22-s0001.tif]

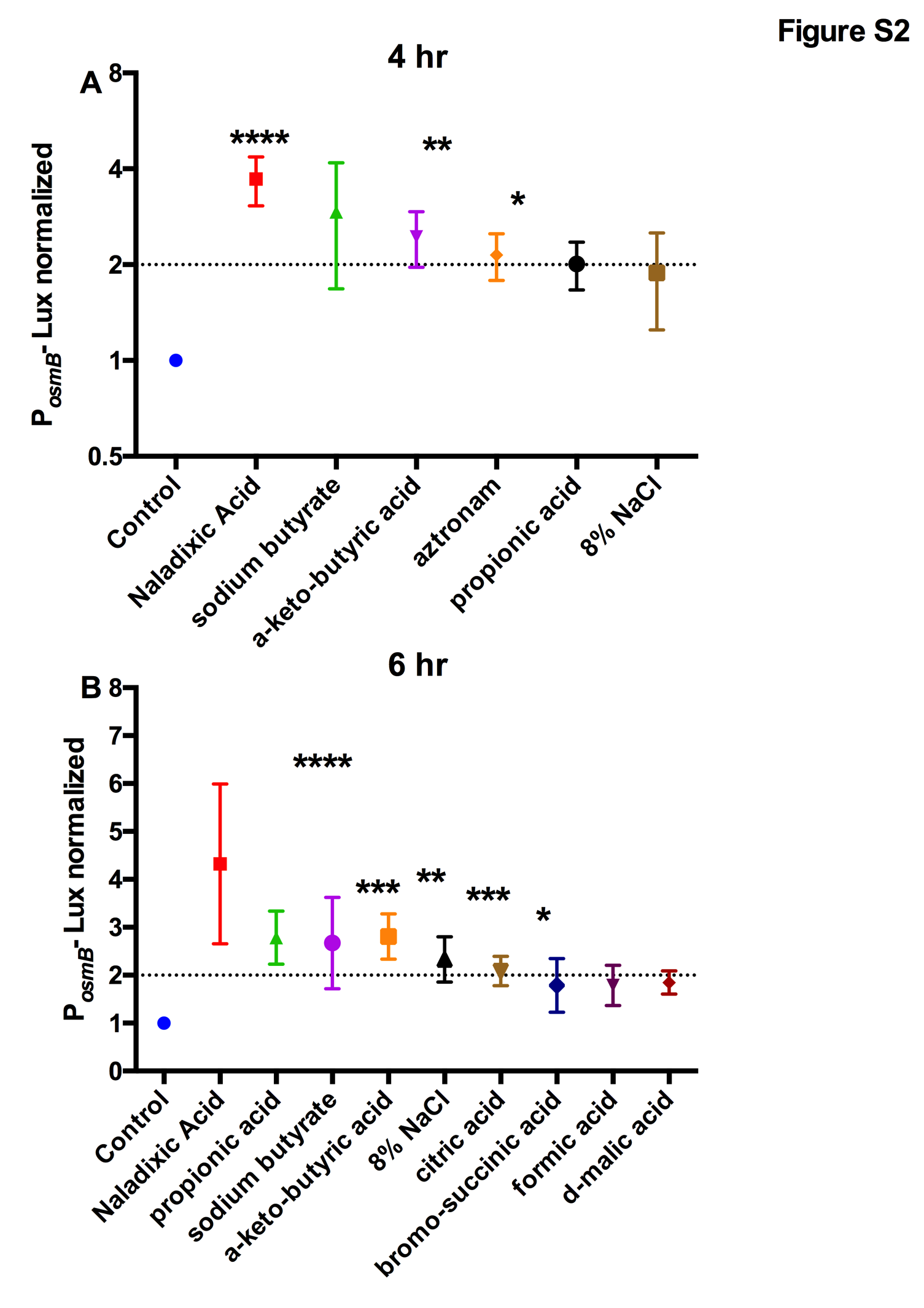

Supplement: FIG S2 [file msphere.00439-22-s0002.tif]

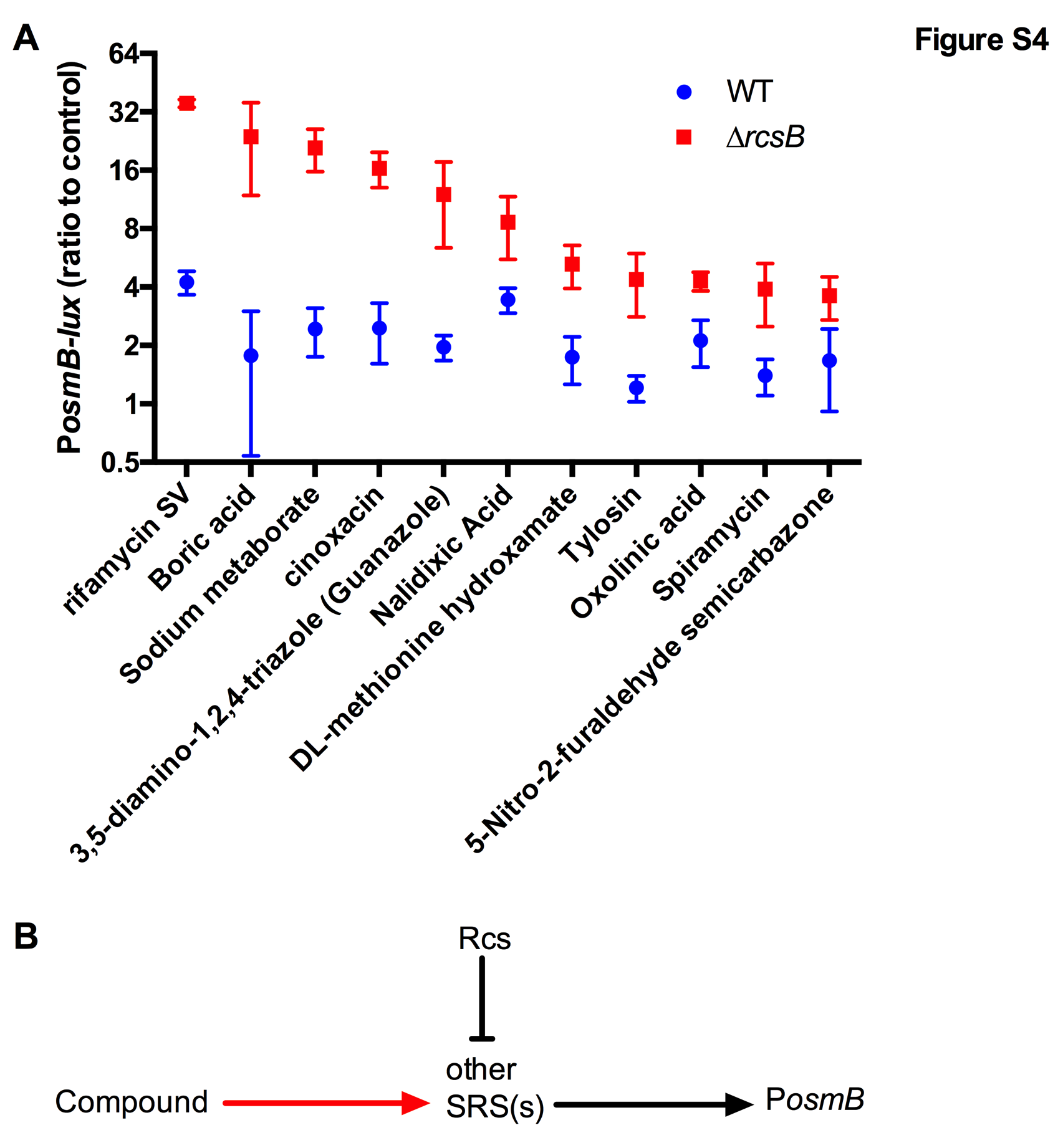

Supplement: FIG S4 [file msphere.00439-22-s0004.tif]

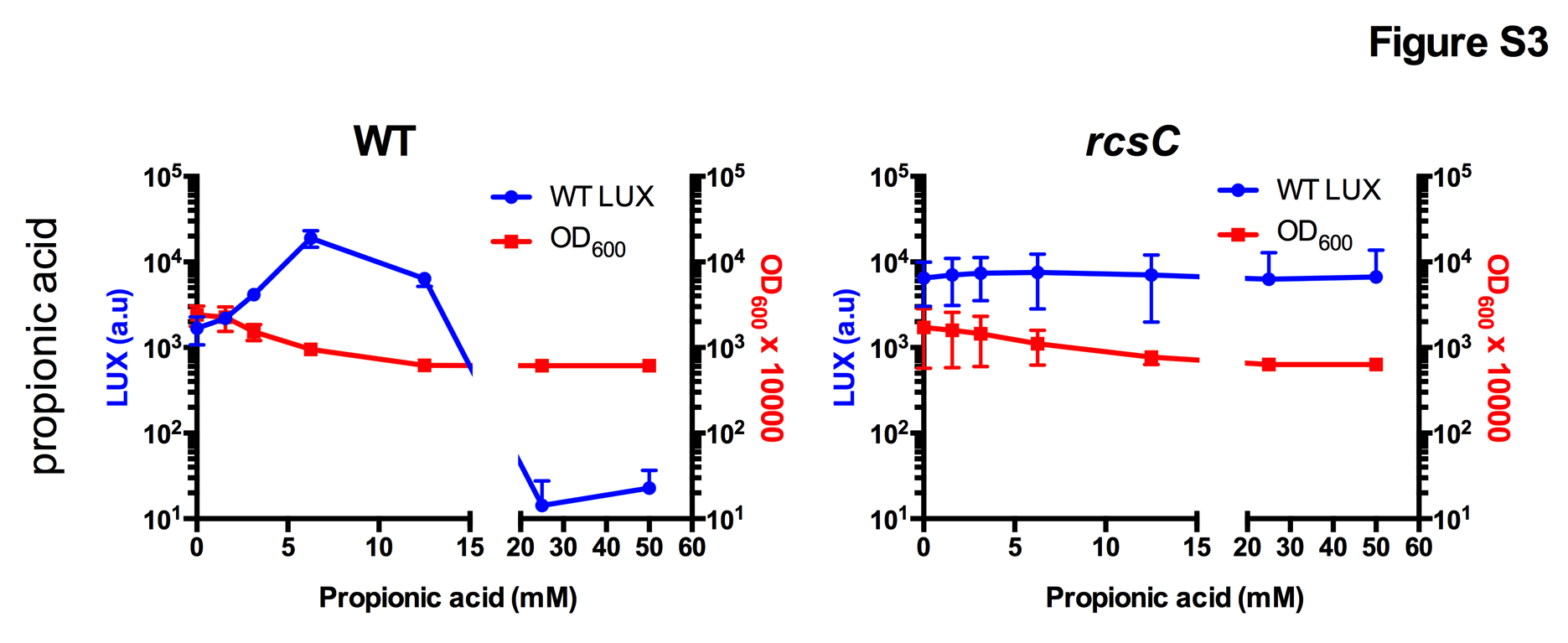

Supplement: FIG S3 [file msphere.00439-22-s0003.tif]

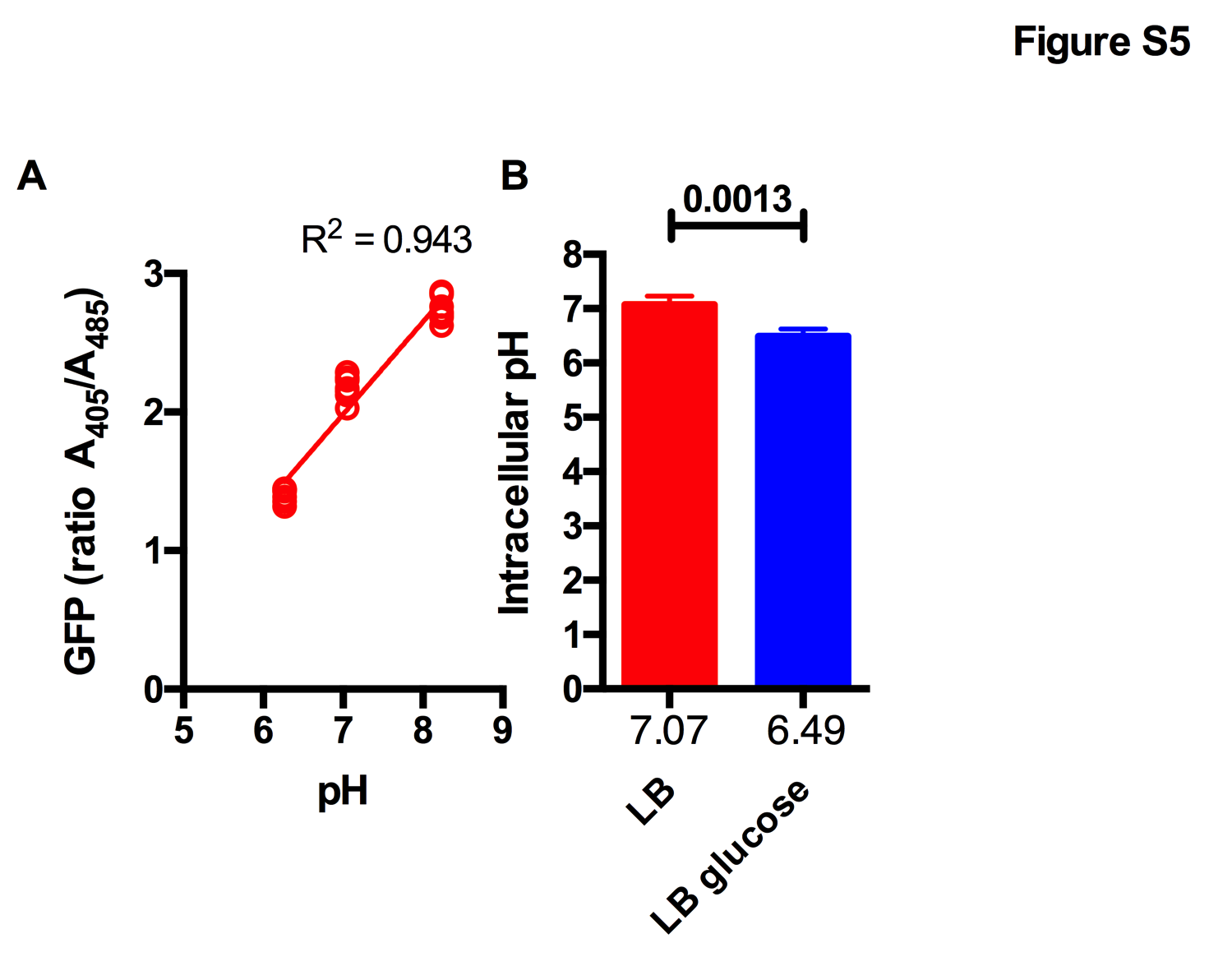

Supplement: FIG S5 [file msphere.00439-22-s0005.tif]

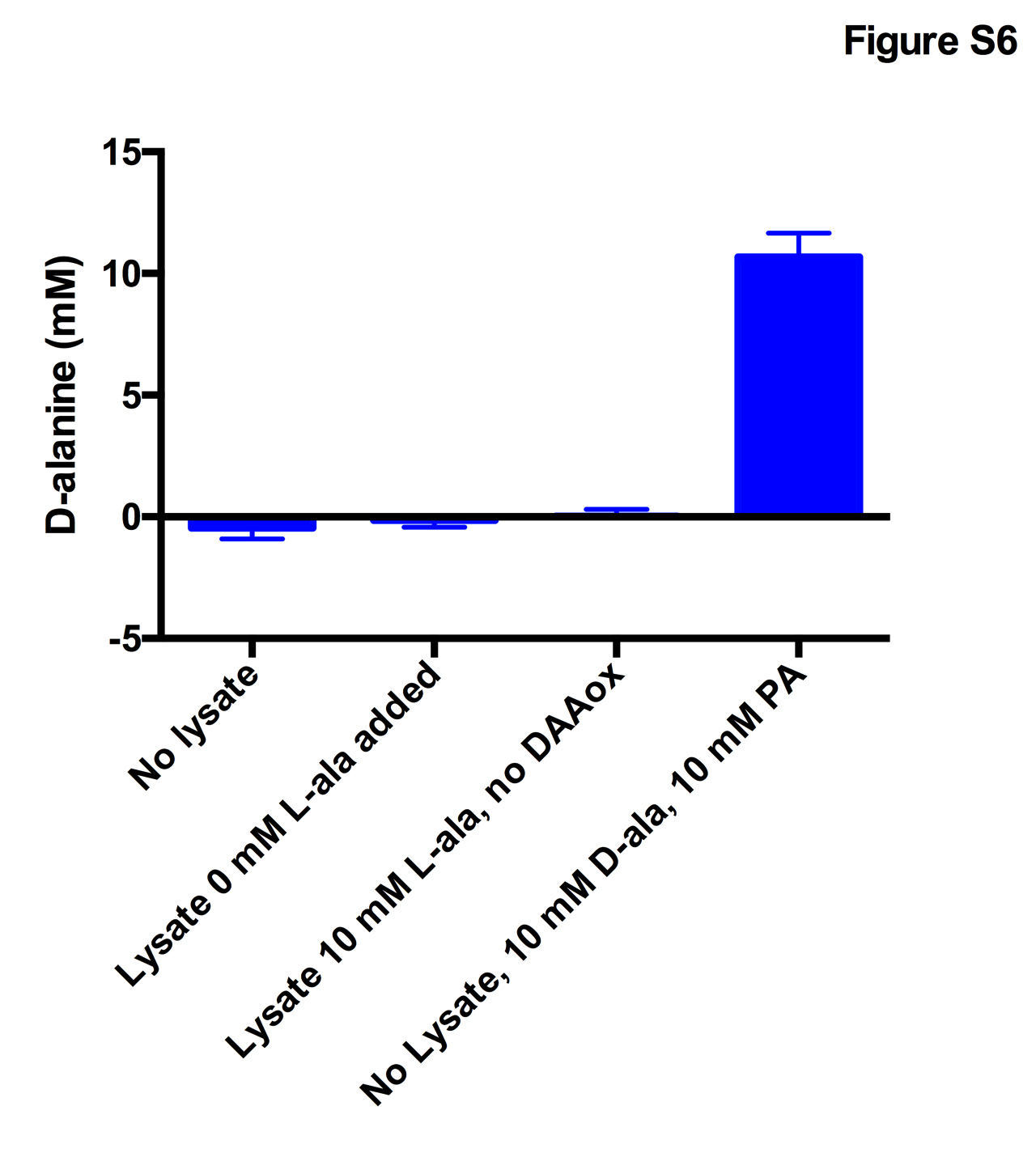

Supplement: FIG S6 [file msphere.00439-22-s0006.tif]
